# Can Smartwatch Prevent Sudden Cardiac Deaths? A Case Of Smartwatch Failure in Arrhythmogenic Right Ventricular Dysplasia

**DOI:** 10.7759/cureus.15904

**Published:** 2021-06-24

**Authors:** Sengottaian Sivakumar, Navdeep Bhatti

**Affiliations:** 1 Anesthesiology, Metropolitan Hospitals, New York, USA; 2 Cardiology, Metropolitan Hospitals, New York, USA

**Keywords:** tachyarrythmias, failed smartwatch, sudden cardiac death, arvd, progressive right ventricular dysfunction

## Abstract

Arrhythmogenic right ventricular dysplasia (ARVD) is caused by mutations in genes coding for components of desmosomes in the myocardium. Mutations in these genes make desmosomes dysfunctional and account for myocyte detachment, followed by inflammation and apoptosis when it encounters undue mechanical stress. This is why ARVD is a common cause of sudden cardiac death in athletes with undiagnosed ARVD, as increased physical activity exacerbates this progression of ARVD and associated arrhythmias. We describe a case of ARVD in a 36-year-old woman who presented with an unusual sensation in her chest due to non-sustaining ventricular tachycardia, which her smartwatch failed to pick up. Many smartwatches use photoplethysmography (PPG) to monitor heart rate (HR). A typical PPG device contains two light sources (green light and infrared) and a photodetector to measure the reflected light, proportional to the beat-to-beat variation in blood volume. HR is then calculated from these variations. In ambulatory settings, smartwatches underestimate HR in most tachyarrhythmias, mainly when the HR is more than 100 beats/min. Patients using smartwatches for ambulatory heart monitoring should know that the absence of an irregular pulse notification does not exclude possible arrhythmias. Management of ARVD is mainly focused on the prevention of syncope and cardiac arrest through antiarrhythmic medications and an implantable cardioverter defibrillator.

## Introduction

Arrhythmogenic right ventricular dysplasia (ARVD) is caused by mutations in genes coding for components of desmosomes in the myocardium. We describe a case of ARVD in a 36-year-old woman who presented with an unusual sensation in her chest due to non-sustaining ventricular tachycardia (VT), which her smartwatch failed to pick up. Wearables can passively measure pulse rate from the wrist using photoplethysmography (PPG). Longitudinal pulse data could be analyzed to assess pulse irregularity and variability to identify potential irregular heart rhythms. However, the utilization of wearables or arrhythmia detection algorithms may not be without risks. Patients using smartwatches for ambulatory heart monitoring should know that the absence of an irregular pulse notification does not exclude possible arrhythmias.

## Case presentation

**Figure 1:**
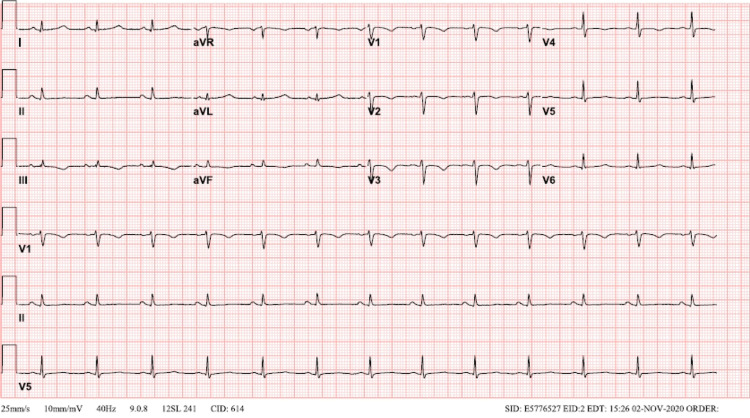
Prolonged QTc (481 ms) with symmetric T wave inversions in the right precordial leads (V1-V3)

A 36-year-old Cantonese-speaking female presented to our institute with a history of intermittent palpitations and dizziness for two days. Her ECG showed prolonged QTc (481 ms) with symmetric T wave inversions in the right precordial leads (V1-V3) (Figure 1). Troponin level was positive at 0.054 ng/mL. She had a similar episode of sudden-onset spontaneous palpitations, which were presumed to be associated with fear and anxiety a month earlier. At that time, she was evaluated in the psychiatry emergency room for generalized anxiety disorder and discharged. There was no known family history of sudden cardiac deaths or strokes. She started to self-monitor using her apple smartwatch (Series 3). However, she could not find anything abnormal whenever she felt palpitations. She then started to use a pulse oximeter at home to monitor her symptoms. Her pulse oximeter showed a heart rate (HR) of more than 200 beats/min during an episode of palpitations and uneasiness in her chest. She went to the hospital with a video showing an HR of more than 200 beats/min (Video 1).

**Video 1:** Pulse oximeter showing heart rate more than 200 beats/min during an episode of ventricular tachycardia

**Figure 2:**
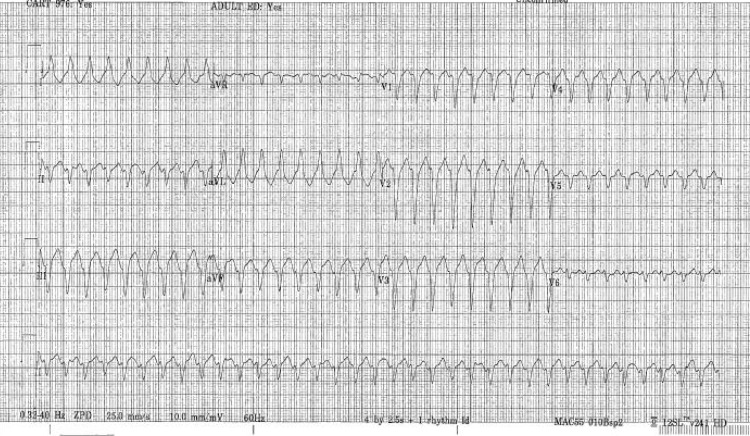
Wide complex ventricular tachycardia with a heart rate of more than 200 beats/min

**Figure 3:**
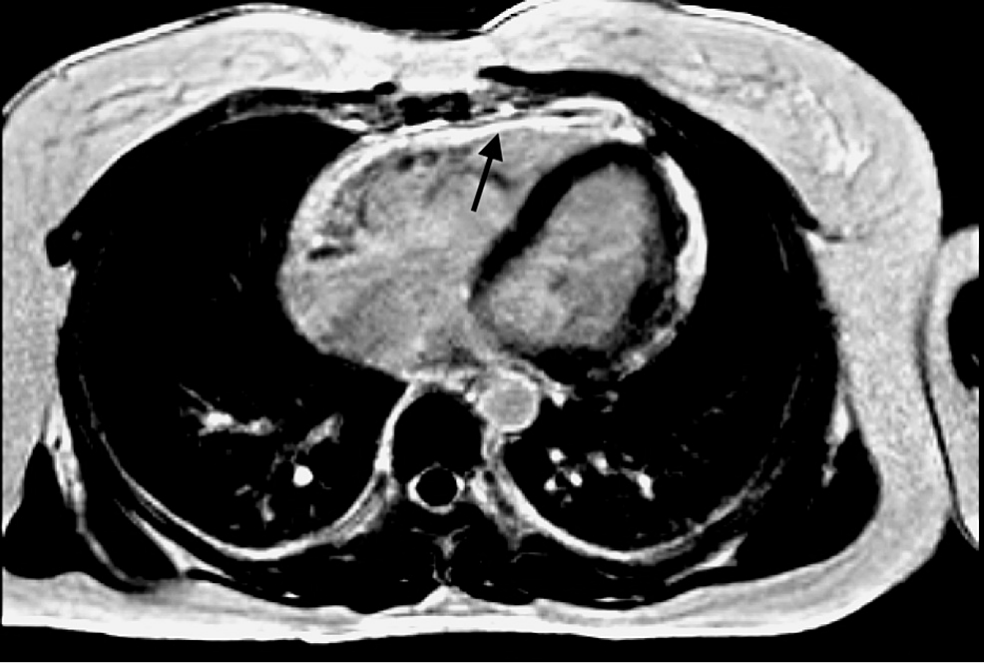
Phase-sensitive inversion recovery late gadolinium enhancement imaging, axial view: there is extensive enhancement of the right ventricle free wall and patchy epicardial enhancement of the left ventricle

**Figure 4:**
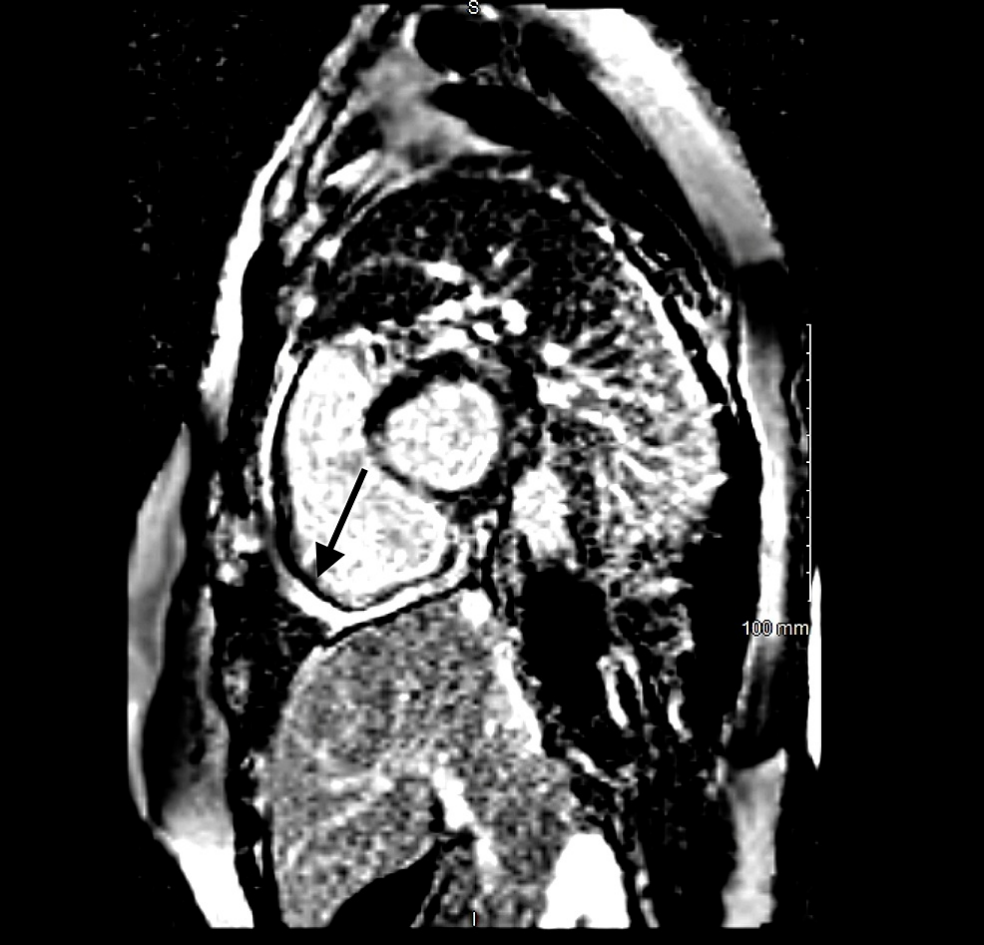
Phase-sensitive inversion recovery late gadolinium enhancement imaging, mid short-axis view: There is extensive enhancement of the right ventricle free wall

Telemetry monitoring showed no abnormalities. Her transthoracic echocardiogram showed dilated and reduced systolic function of the right ventricle (RV) with an apical echogenic tissue and abnormal RV free wall motion. We scheduled her for cardiac magnetic resonance imaging (CMRI) and discharged her with an ambulatory 30-day event monitor. Two days after her discharge, she presented again to the emergency room with shortness of breath and palpitations that started while bending to lift a heavy object. She denied excess caffeine intake, syncope, chest pain, or recreational drug usage. Her ECG showed wide complex VT with a rate of more than 200 beats/min (Figure 2). Labs were notable for positive troponin 0.016 ng/mL, pro B-type natriuretic peptide 143 pg/mL, lactate of 2.8 mmol/L, Mg 1.7 mEq/L, and Hg 11.2 g/dL (mean corpuscular volume 82 fL). Her rhythm converted to normal sinus rhythm with 50 mg of oral metoprolol and 2 g of intravenous magnesium. Her CMRI showed dilatation of RV with moderately decreased systolic function and multiple areas of thinning and severe hypokinesis of RV with extensive fibrosis and fibro-fatty infiltration, which were consistent with ARVD (Figures 3, 4). Some areas of fibrosis in the left ventricle wall were also noted. Ejection of RV was 34%. Due to multiple episodes of non-sustained VT on 30-day cardiac event monitoring, metoprolol was discontinued, and the patient was started on sotalol. An electrophysiology consult was requested for the placement of an automatic implantable cardioverter defibrillator (AICD).

 

The patient was monitored with telemetry for two days after placing AICD. Telemetry revealed predominantly normal sinus rhythm with occasional premature ventricular contractions of two dominant morphologies and multiple runs of non-sustained VT. Genetic testing showed missense mutations in DSG2 and SCN5A genes associated with the autosomal-dominant version of ARVD. 

## Discussion

There has been much debate about the accuracy of ambulatory ECG monitoring with smartwatches. Many smartwatches use PPG to monitor HR. A typical PPG device contains two light sources (green light and infrared) and a photodetector to measure the reflected light, proportional to the beat-to-beat variation in blood volume. HR is then calculated from these variations. In ambulatory settings, smartwatches underestimate HR in most tachyarrhythmias, mainly when the HR is more than 100 beats/min [1-3]. In one study, all wearable devices tested were poor at detecting short episodes of SVT, particularly for episodes less than 60 s in duration [2]. Device algorithms filter out the transient increase in HR, treating it as noise distorting the actual signal [2]. Our patient initially relied on an Apple smartwatch (Series 3), but as it did not show any changes during her symptoms, she started using a handheld pulse oximeter. The pulse oximeter reliably revealed an HR of more than 200 beats/min during the event (Video 1). Usually, handheld pulse oximeters accurately estimate HR at rest and during submaximal exercise (i.e., at a rate of <155 beats/min or <89% of maximum) but tend to underestimate HR during heavy exercise [4]. ARVD is a disease caused by mutations in genes coding for components of desmosomes in myocardial skeletal muscle. The desmosome is the mechanical bridge that links one myocardial cell to the next. Mutations in these genes make desmosomes dysfunctional and account for myocyte detachment, followed by inflammation and apoptosis when it encounters undue mechanical stress. This is why ARVD is a common cause of sudden cardiac death in athletes with undiagnosed ARVD, as increased physical activity exacerbates this progression of ARVD and associated arrhythmias [5]. More than 50% of cases are familial and inherited predominantly in an autosomal-dominant fashion, with variable expressivity and incomplete penetrance. In recessive forms (Naxos disease, Carvajal syndrome), cutaneous manifestations are apparent. Both forms of the disease usually manifest as palpitations and syncope commonly observed during the third decade of life [6]. In our patient, sequence analysis deletion/duplication testing showed missense mutations in DSG2 and SCN5A genes coding for desmosomes. SCN5A gene codes for sodium channels and this gene mutation is found only in 2% of ARVD cases [7]. It is generally characterized by progressive right ventricular (RV) dysfunction and ventricular arrhythmias, leading to sudden cardiac death. ARVD can also involve the left ventricle and cause left-sided heart failure in advanced stages of the disease [5]. Histologically, fibro-fatty tissue replaces ventricular myocardium with perpetual dilation and systolic dysfunction. VF can occur during myocardial cell death, whereas reentrant VT is related to fibro-fatty scar tissue. Scarring initially produces regional wall motion abnormalities but later may involve the free wall and become global, producing ventricular dilation. ECG findings include symmetrically inverted T waves and epsilon waves in the right precordial leads (V1, V2, and V3), possibly due to slow depolarization of the affected RV myocardium. According to standardized diagnostic criteria proposed by an international task force [8], the diagnosis of ARVD is based on the presence of major and minor criteria encompassing ECG, arrhythmic, RV morphological, functional, and histopathological factors. The diagnosis is achieved in the presence of two major criteria or one major plus two minor or four minor criteria from different groups [8]. In our patient, the definite diagnosis of ARVD can be made as she has more than two major criteria, CMRI finding of RV dyskinesia with foci of fat in RV myocardium, reduced systolic function of RV, T wave inversions in anterior leads, and finally witnessed VT in a left bundle branch block pattern. The presence of late gadolinium enhancement on CMRI, which correlates with the fibro-fatty changes, is a diagnostic feature of ARVD [9]. Moreover, myocyte apoptosis in patients with ARVD and increased myocardial stretch through cytokines released by adipocytes may lead to an elevated serum troponin measurement [10]. Troponin levels were elevated on several occasions in our case. Management of ARVD is mainly focused on the prevention of syncope and cardiac arrest through antiarrhythmic medications and an ICD. CMRI has played a significant role in diagnosing ARVD with a reasonably high sensitivity and specificity. Usually, an endomyocardial biopsy is not required to diagnose ARVD due to patchy involvement of the myocardium. Young age onset (<40 years), positive family history, QRS dispersion ≥40 ms, T-wave inversions beyond lead V1, left ventricular involvement, VT, syncope, and previous cardiac arrest are considered the major determinants for adverse prognosis, including sudden cardiac death [11]. The expert consensus is that ICD therapy is indicated for primary prevention for high-risk individuals and secondary prevention for those with a prior history of sustained VT [12]. Sotalol is considered an effective antiarrhythmic agent in the treatment of ARVD-associated arrhythmias. In addition to beta-blocker activity, it also inhibits the efflux of potassium ions, which increases the time before another electrical signal can be generated in ventricular myocytes [13]. Unfortunately, though, the mainstay of arrhythmia prevention relies on AICD device insertion, which also carries a risk of inappropriate shocks. In our patient, cardiac event monitoring revealed several episodes of NSVT before AICD implantation, which prompted the initiation of sotalol treatment.

## Conclusions

Patients using smartwatches for ambulatory heart monitoring should be aware that the absence of an irregular pulse notification does not exclude possible arrhythmias. The algorithm is designed to reduce the chances of incorrect HR readings; a common strategy is to stop recording when high levels of motion interference are detected. Unfortunately, this means that there is a high chance that a true cardiac arrhythmia will be interpreted as motion interference. It is essential to understand the limitations of these technologies to avoid inappropriate reliance on them for diagnostic purposes.
